# Transcriptome Profiling Reveals the Antitumor Mechanism of Polysaccharide from Marine Algae *Gracilariopsis lemaneiformis*

**DOI:** 10.1371/journal.pone.0158279

**Published:** 2016-06-29

**Authors:** Yani Kang, Hua Li, Jun Wu, Xiaoting Xu, Xue Sun, Xiaodong Zhao, Nianjun Xu

**Affiliations:** 1 Key Laboratory of Marine Biotechnology of Zhejiang Province, School of Marine Sciences, Ningbo University, Ningbo, Zhejiang, P.R. China; 2 School of Biomedical Engineering, Bio-ID Center, Shanghai Center for Systems Biomedicine, Shanghai Jiao Tong University, Shanghai, P.R. China; University of Pittsburgh School of Medicine, UNITED STATES

## Abstract

Seaweed is one of the important biomass producers and possesses active metabolites with potential therapeutic effects against tumors. The red alga *Gracilariopsis lemaneiformis* (*Gp*. *lemaneiformis*) possesses antitumor activity, and the polysaccharide of *Gp*. *lemaneiformis* (PGL) has been demonstrated to be an ingredient with marked anticancer activity. However, the anticancer mechanism of PGL remains to be elucidated. In this study, we analyzed the inhibitory effect of PGL on the cell growth of 3 human cancer cell lines and found that PGL inhibited cell proliferation, reduced cell viability, and altered cell morphology in a time- and concentration-dependent manner. Our transcriptome analysis indicates that PGL can regulate the expression of 758 genes, which are involved in apoptosis, the cell cycle, nuclear division, and cell death. Furthermore, we demonstrated that PGL induced apoptosis and cell cycle arrest and modulated the expression of related genes in the A549 cell line. Our work provides a framework to understand the effects of PGL on cancer cells, and can serve as a resource for delineating the antitumor mechanisms of *Gp*. *lemaneiformis*.

## Introduction

Cancer is among the leading causes of morbidity and mortality worldwide, and effective antitumor drugs are urgently needed to control human malignancies [1]. However, many cancer cells show significant resistance to chemotherapeutic drugs, and almost all of the chemotherapy drugs on the market cause serious side effects. Therefore, there is an urgent need for new alternative therapeutic agents, such as biological macromolecules, which may be nontoxic or have fewer side effects to fight cancer [2].

In the past decade, an increasing body of evidence has revealed that marine products could serve as antitumor agents and play a preventive role in controlling tumors, underlining their potential in the discovery of new pharmaceuticals [3]. Marine organisms are well known to provide a wide range of natural antitumor compounds, and seaweed is one of the important biomass producers and possesses active metabolites with potential therapeutic effects against tumors, such as polysaccharides and polysaccharide-protein complexes, which possess antitumor activities or can increase the efficacy of conventional chemotherapy drugs [4–6]. Based on the encouraging observations to date, a great deal of effort has been focused on discovering antitumor polysaccharides for the development of effective therapeutics for various tumors. The red alga *Gracilariopsis lemaneiformis* (*Gp*. *lemaneiformis*) has active metabolites and ingredients with antitumor, antiviral, antioxidant, hypotensive, and hypolipidemic effects [7–9]. The polysaccharide of *Gp*. *lemaneiformis* (PGL) has been demonstrated to be an ingredient with marked antitumor activity and is an ideal potential nontoxic preventive agent [9]. In previous studies, we confirmed that PGL could significantly inhibit the proliferative activity and alter the cell morphology of lung tumor cells [10]. However, the underlying mechanism remains to be elucidated.

The current study is the first to perform genome-wide transcriptome analysis to reveal the antitumor mechanism of PGL. We measured the effect of PGL on cellular growth and viability in the cervical carcinoma cell line HeLa, the lung cancer cell line A549, and the human gastric cancer cell line MKN45, and observed the most significant anticancer effect in A549 cells. Furthermore, we performed transcriptome analysis to identify the genes whose expression is modulated by PGL using RNA sequencing (RNA-Seq). Gene ontology analysis of differentially expressed genes indicated that the biological processes of the cell cycle, apoptosis, nuclear division, and translation could be modulated by PGL. In particular, we found that PGL significantly modulates the expression of apoptosis- and cell cycle-related genes. In addition, we demonstrated that PGL induces apoptosis and cell cycle arrest using Annexin V-FITC and propidium iodide (PI) fluorescence-activated cell sorting (FACS) analysis, flow cytometry, and real-time quantitative PCR (RT-qPCR). Our study provides new insight into the understanding of PGL anticancer mechanisms.

## Materials and Methods

### Ethics Statement

*Gp*. *lemaneiformis* 981 was collected from the seashores of Wenzhou, Zhejiang Province of China (27° 52’ N, 120° 36 E) in October 2014; this location is neither privately owned nor a protected place. As a normal red alga, no specific permits are required at this point for studies on *Gp*. *lemaneiformis* 981.

### PGL Extraction and Purification

*Gp*. *lemaneiformis* was washed several times with distilled water, and then vacuum freeze-dried. The polysaccharides were extracted from *Gp*. *lemaneiformis* and purified as described in our previous study [10, 11]. Briefly, the powdered *Gp*. *lemaneiformis* was extracted with 90-fold volumes of distilled water for 4 h at 80°C. After centrifugation to remove residues, the supernatant was concentrated in a vacuum rotary evaporator. The concentrated solution was precipitated and then resolved in warm water. Proteins were removed using the Savage method (Chloroform: n-butyl alcohol = 4:1). There is an obvious protein precipitation after washed three times with 16 ml chloroform and 4 ml n-butyl alcohol, the upper solution was taken and washed twice with anhydrous ethanol precipitation, then added 5 ml of distilled water to dissolve the precipitation, that is the crude polysaccharide solution. The supernatant of polysaccharides was dialyzed through a dialysis membrane with a pore diameter of 3500 D in distilled water for 48 h and then vacuum freeze-dried. Finally, the polysaccharides were purified using diethylaminoethyl-cellulose (DEAE-C; Sigma-Aldrich, St. Louis, MO, USA) with chloride sodium (Sigma-Aldrich). Each purified fraction had only one main peak, the main peaks were collected, concentrated, lyophilized and marked as PGL for following assays (S1 Fig).

### Cell Culture and PGL Treatment

The human gastric cancer cell line MKN45, non-small cell lung cancer (NSCLC) cell line A549, and cervical carcinoma cell line HeLa were purchased from the Chinese Academy of Sciences Committee on Type Culture Collection Cell Bank (Shanghai, China). Cells were cultured in Roswell Park Memorial Institute (RPMI)-1640 medium (Invitrogen Corp., Waltham, MA, USA) supplemented with 10% fetal bovine serum (FBS; Gibco, Carlsbad, CA, USA), 100 units/mL penicillin (Sigma-Aldrich), and 100 μg/mL streptomycin (Sigma-Aldrich) at 37°C in a humidified incubator containing 5% CO_2_. For PGL treatment and antitumor analysis, cells were seeded into a 6-well culture plate at a density of 5 × 10^5^ cells/well and treated with serial concentrations of PGL in a humidified atmosphere with 5% CO_2_ for 24, 48, and 72 h, respectively. A concentration of 6 μg/mL of the common antitumor agent cisplatin (DDP) was selected for our protocol since this dose caused the death of about 50% of A549 cells.

### Cell Viability Analysis

To investigate the effect of PGL on cancer cell viability and survival rate, cells were subjected to the trypan blue exclusion assay and the Cell Counting Kit-8 (CCK-8) colorimetric method. For CCK-8 detection, viable cells treated with different concentrations of PGL extract were evaluated by CCK-8 (Dojindo Laboratories, Kumamoto, Japan). Briefly, 2 x 10^3^ cells were seeded in each well of 96-well plates with 200 μL growth medium. After overnight incubation, serial concentrations of PGL (0, 5, 10, 20, 30, 40, 50, 60, 80, and 100 μg/mL) were added and the cells were incubated in a humidified atmosphere with 5% CO_2_ for 24, 48, and 72 h. Then, the PGL-containing medium in each well was replaced with 100 μL fresh medium with 10 μL CCK-8 solution. After incubation at 37°C for 3 h, the plate was read at OD 450 nm using a spectrophotometric plate reader (BioTek Instruments, Inc., Winooski, VT, USA). The absorbance of the control group (0 μg/mL) was considered to be 100%. The results were tested by three independent experiments. For trypan blue staining, 24 h after treatment the cell suspension was mixed with 0.4% trypan blue solution (Beijing Solarbio Science & Technology Co., Ltd., Beijing, China) in a 1:1 ratio. After 3 min incubation at room temperature, the mixture was loaded onto one chamber of a Neubauer hemocytometer and the squares of the chamber were observed under a light microscope. The viable/live (clear) and nonviable/dead (blue) cells were counted and the viability was calculated using the formula (number of live cells counted/total number of cells counted) × 100.

### Negative and Positive Controls

The above results of the CCK-8 assay revealed that purified PGL can reduce cellular viability and cell growth in lung cancer, gastric cancer, and cervical cancer cells, and particularly in A549 lung cancer cells. Therefore, we further studied the antitumor mechanism of PGL in the A549 lung cancer cell line. For a negative control, the cell viability of normal human lung cells (BEAS-2B) and human lung fibroblasts (MRC-5) was investigated by the CCK-8 assay after the normal control cells were treated with PGL for 24, 48, and 72 h. For a positive control, the common antitumor agent cisplatin (DDP) was compared with PGL to detect their cytotoxicity and effectiveness by using CCK-8. After cell adherence, the A549 cells were incubated with dimethyl sulfoxide (DMSO; control) or DDP (3 μg/mL) or 50 μM PGL for 24, 48, and 72 h and then collected for the CCK-8 assay as described above. The dosage of PGL (50 μM) is the fifty percent inhibitory concentration (IC_50_) on A549 cells for 48 h according to the results of the CCK-8 assay. The dosage of DDP (6 μM) is based on the results of a previous study [12].

### Cell Apoptosis Analysis

Detection of phosphatidylserine (PS) and determination of membrane integrity were performed using the Annexin V/PI Apoptosis kit (Zoman Biotechnology, Beijing, China). Briefly, 1 x 10^5^ A549 cells were seeded in each well of 6-well plates. For fluorescence imaging, the control (without PGL) and PGL-treated (50 μM) cells stained with Annexin V/PI for 24, 48, and 72 h were observed by fluorescence microscopy (Model Ti-E; NIS4.0; Nikon, Fukasawa, Japan). The other portion of the A549 cells was harvested using trypsin without EDTA (Life Technologies Corp., Carlsbad, CA, USA) and washed twice with phosphate-buffered saline (PBS), then stained with Annexin V/PI and analyzed by flow cytometry (BD LSRFortessa; BD Biosciences, San Jose, CA, USA).

### Cell Cycle Analysis

Briefly, 1 x 10^6^ PGL-treated cells were harvested using trypsin without EDTA (Life Technologies Corp.), and washed twice with PBS. The cells were fixed overnight with cold 70% ethanol, and then stained with a PI solution consisting of 20 μg/mL PI, 0.1% Triton X-100 (Sigma-Aldrich), and 100 μg/mL RNase A (Fermentas International Inc., Burlington, ON, Canada) for 30 min at 37°C in the dark. The relative proportions of cells in the G1, S, and G2/M phases of the cell cycle were analyzed using the flow cytometry data (BD Biosciences).

### RNA-Seq Library Construction and Data Accession

Total RNA was extracted using Trizol (Life Technologies Corp.), and further treated with DNase to remove genomic DNA contamination. Isolation of mRNA was performed using the Oligotex mRNA Mini Kit (Qiagen GmbH, Hilden, Germany) and the mRNA was then used for RNA-Seq library construction with the NEB Next Ultra Directional RNA Library Prep Kit for Illumina (New England Biolabs, Ipswich, MA, USA); the products were then subjected to Illumina sequencing. Raw sequencing data of both the control and PGL-treated groups were submitted to the National Center for Biotechnology Information (NCBI) Sequence Read Archive (SRA; http://www.ncbi.nlm.nih.gov/sra) database (accession number: SRP064837).

### Analysis of Differentially Expressed Genes

The Trimmomatic software was introduced to ensure the quality of sequencing reads to be mapped. The raw reads are mapped to reference genome (hg19) with TopHat v2.1.0 [13]. The gene expression level is measured by reads per kilobase of transcript per million reads mapped (RPKM) and calculated with Cufflinks v2.2.1 [14], which is used to perform the differential gene expression analysis, and genes with a false discovery rate (FDR) adjusted *p*-value less than 0.05 are regarded as significantly differentially expressed genes.

### Functional Annotation and Pathway Analysis

The Database for Annotation, Visualization and Integrated Discovery (DAVID) bioinformatics resource was used to annotate gene functions and pathways [15]. Functional enrichment analyses were performed using online tools embedded in DAVID 6.7 (https://david.ncifcrf.gov/). As a representative subset of the GO terms in the underlying Gene Ontology Annotation (GOA) database, the network system of the apoptosis- and cell cycle-related genes was further analyzed using a simple clustering algorithm that relies on semantic similarity measures [16]. REViGO is freely available at http://revigo.irb.hr/.

### Quantification of mRNA Level

The quantification of mRNA levels of apoptosis-related genes was evaluated using RT-qPCR. Total RNA was extracted using Trizol (Life Technologies Corp.) and subjected to reverse transcription with random primers using SuperScript III (Life Technologies Corp.) to obtain cDNAs. RT-qPCR was performed using a SYBR Green PCR Master Mix (DBI Bioscience, Shanghai, China) according to the manufacturer’s protocol. The primer sequences were used for real-time PCR (S5 Table). Threshold cycle (Ct) values were automatically calculated by the Applied Biosystems StepOnePlus system (Applied Biosystems Inc., Foster City, CA, USA). The PCR conditions were as follows: 95°C for 10 min, followed by 40 cycles of 95°C for 15 sec and 60°C for 1 min. The intensity of each gene was normalized by *GAPDH* expression. According to the ΔΔCt method, differential expression was calculated as a ratio of the expression levels of target genes in PGL-treated and -untreated cells.

### Statistical Analysis

Statistical analysis was performed using R (https://cran.r-project.org/index.html) and SPSS 12 software (SPSS Inc., Chicago, IL, USA). Cell survival rates are presented as the mean ± standard deviation (SD) of at least five independent experiments. The Student *t* test was used to determine whether there was a significant difference between experimental variables. A *p*-value < 0.05 was considered statistically significant.

## Results

### PGL Inhibits Cell Proliferation and Reduces Cell Viability

**Fig 1:**
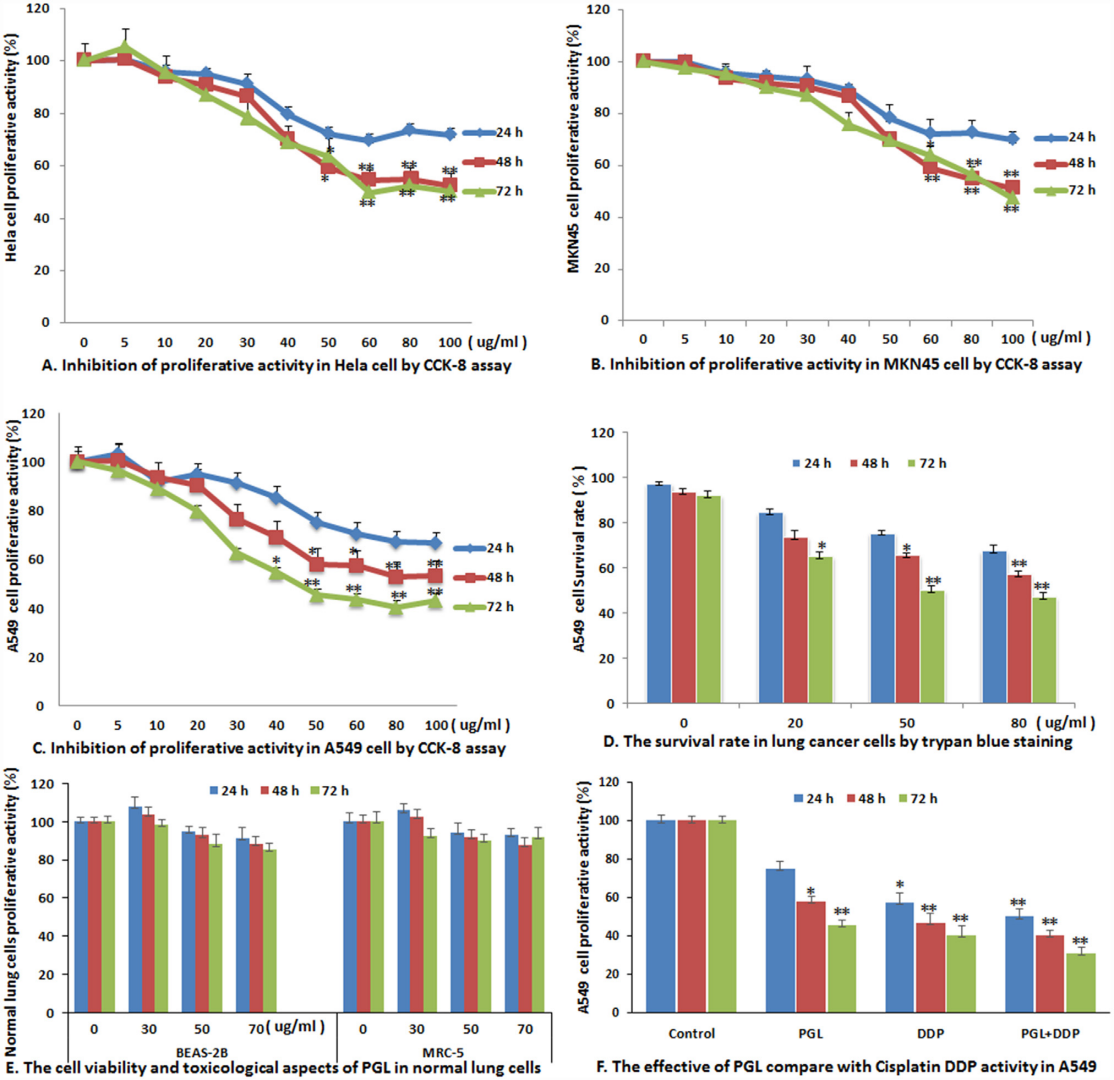
Inhibitory effect of PGL on proliferative activity in different cancer cells. (A-C) The proliferation of various types of cancer cells cultured with different dosages of PGL was evaluated by using the Cell Counting Kit-8 (CCK-8) assay, including the cervical carcinoma cell line HeLa (A), the gastric cancer cell line MKN45 (B), and the lung cancer cell line A549 (C). Cancer cells were treated with PGL (0–100 μg/mL) for 24, 48, and 72 h, respectively, and examined for viability using the CCK-8 assay (n = 3); (D) PGL reduced the survival rate in A549 cells by trypan blue staining (n = 5); (E) The cell viability and toxicological aspects of PGL in normal human lung cells (BEAS-2B) and human lung fibroblasts (MRC-5) were investigated after PGL treatment for 24, 48, and 72 h using the CCK-8 assay (n = 3); (F) The effectiveness of PGL compared with the usual antitumor agent cisplatin (DDP). The A549 cells were incubated with 6 μM DDP or 50 μM PGL for 24, 48, and 72 h and then analyzed with the CCK-8 assay. (* *P* < 0.05, ** *P* < 0.01).

We first examined the effect of PGL on cancer cells. Three cancer cell lines were treated with PGL extracts at various concentrations (0, 5, 10, 20, 30, 40, 50, 60, 80, and 100 μg/mL) for different effect times (24, 48, and 72 h, respectively). The proliferation of cells cultured with different dosages of PGL was evaluated using the CCK-8 assay. We found that PGL extracts demonstrated growth inhibitory effects on HeLa, MKN45, and A549 cells in a dose-dependent manner (Fig 1A–1C), with the most remarkable effect on A549 cells. The IC_50_ was approximately 50 μg/mL at 48 h in A549 cells. Furthermore, the results of cell viability were consistent with the cell proliferation ability by trypan blue staining (Fig 1D). The cytotoxicity of PGL in normal human lung cells (BEAS-2B) and human lung fibroblasts (MRC-5), as normal control cells, demonstrates that PGL induces almost no cytotoxicity in BEAS-2B and MRC-5 cells according to the CCK-8 assay after treatment with different doses of PGL for 24, 48, and 72 h (Fig 1E). The common antitumor agent DDP is widely used as the first-line chemotherapeutic agent in treatment of human NSCLC [12]. Therefore, to compare the effectiveness of DDP vs. PGL, A549 cells were incubated with 6 μM DDP or 50 μM PGL for 24, 48, and 72 h and then collected for the CCK-8 assay (Fig 1F). The results indicate that PGL can not only inhibit cell proliferation, but can also enhance the effect of DDP.

### PGL Modulates Transcriptome Analysis

**Fig 2:**
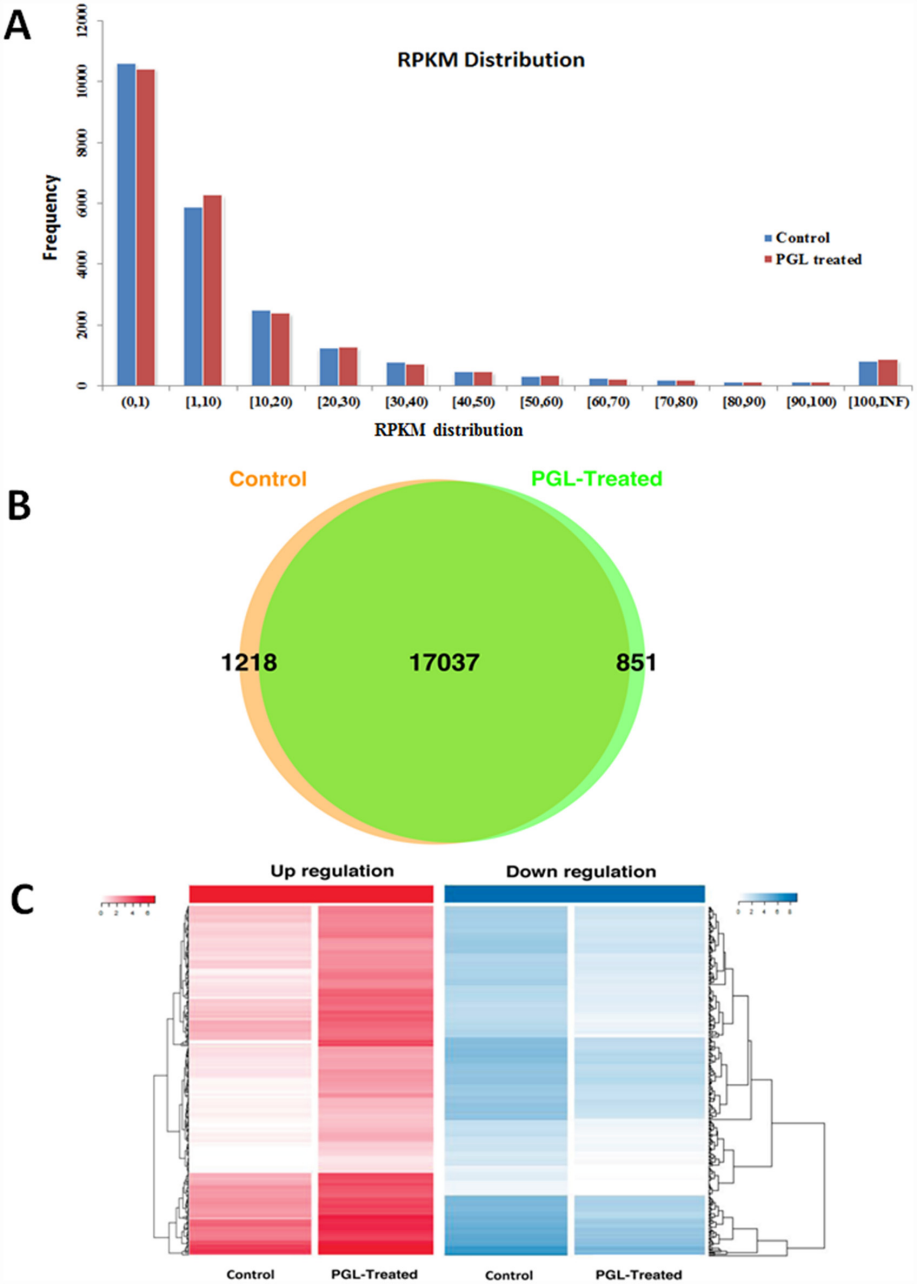
Screening results of the differentially expressed genes in PGL-treated A549 cells and control cells. (A) Distribution of gene expression values; (B) Comparison between the expressed genes of the PGL-treated group and the control group; (C) Heat maps for up- and downregulated genes. The expression level of each gene is indicated by the color. Color bars above the heat map represent sample groups: red is for upregulated genes and blue is for downregulated genes.

To explore the transcriptional regulatory mechanisms of the antitumor effects of PGL, we performed transcriptome analysis and compared the expression pattern on a genome-wide scale in PGL-treated A549 cells with the untreated cells. For RNA-Seq analysis, we generated a total of 21,572,850 reads in the PGL-treated group and 23,243,742 reads in the control group. More than 80% were uniquely mapped to the human genome, which correspond to 17,901 and 18,269 expressed genes, respectively (Table 1). Then we used Cuffdiff to quantify gene expression levels with RPKM, and show the difference between the gene expression levels of the two groups in Fig 2A and S1 Table. We found that 851 genes and 1,218 genes were expressed only in the PGL-treated group and the control group, respectively (Fig 2B). Furthermore, with a *p*-value < 0.05 and fold change (FC) > 2 as the threshold, we identified a total of 758 differentially expressed genes, of which 48.2% (365) were downregulated and 51.8% (393) were upregulated (S2 Table). These 758 up- and downregulated genes with differential expression were demonstrated by Heatmap. For each gene, expression signals were normalized to obey a standard normal distribution (Fig 2C).

**Table 1:** Mapping results of the RNA-Seq data.

	Clean Rds	Unique mapping	Mapping	Total gene	Mapping gene	Mapping gene
	num	Rds num	rate	Num	num	Rate
**Control**	23,243,742	19,747,913	84.9%	23,284	18,255	78.5%
**PGL**	21,572,850	17,353,289	80.4%	23,284	17,888	76.9%

### Gene Ontology Analysis of Differentially Expressed Genes

**Fig 3:**
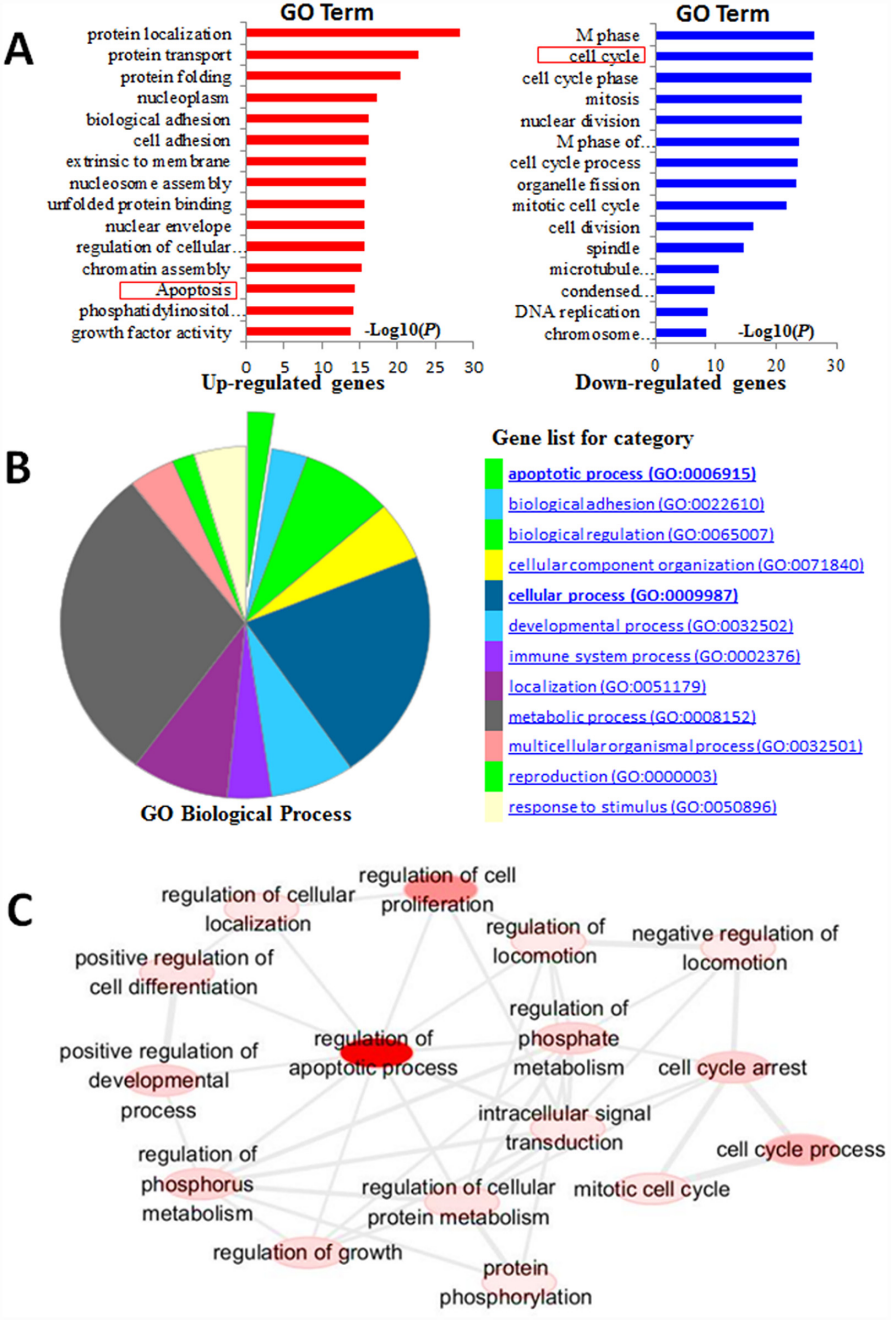
Functional classification and pathway analysis of differentially expressed genes. (A) Gene Ontology (GO) analysis of differentially expressed genes; (B) GO Biological Process of differentially expressed genes in PGL-treated A549 cells compared to control cells; (C) The “interactive graph” view of REViGO. Bubble color indicates the user-provided *p*-value; bubble size indicates the frequency of the GO term in the underlying GOA database. Highly similar GO terms are linked by edges in the graph, where the line width indicates the degree of similarity. The initial placement of the nodes is determined by a “force-directed” layout algorithm that aims to keep the more similar nodes closer together.

To understand the biological significance of these differentially expressed genes, DAVID was used to perform Gene Ontology (GO) analysis and annotate genes. We first investigated the enrichment of GO categories of these genes. Then we further used DAVID to perform KEGG-based annotation and executed pathway enrichment analysis of all the differentially expressed genes using DAVID. The top 15 enriched pathways of downregulated genes included cell cycle, nuclear division, DNA replication, and mismatch repair, which closely affected cell survival and proliferation. The most enriched terms among the upregulated genes were involved in apoptosis, cell death, and protein transport (Fig 3A). The upregulated pathway enrichments were involved in apoptosis, cell adhesion, and biological adhesion (S3 Table). On the other hand, downregulated genes focused on the cell cycle, nuclear division, and translation, among others (S4 Table). All these effects focused on pathways of apoptosis (S2 Fig), cell cycle (S3 Fig), and DNA replication (S4 Fig), which were involved in cell proliferation ability and cell viability. In particular, we also found by GO biological process and pathway analysis that PGL could induce apoptosis andcell adhesion in lung cancer (Fig 3B). These results indicate that PGL might modulate A549 cell biological processes through inhibition of the cell cycle, translation, and nuclear division; activation of apoptosis and biological adhesion; and regulation of immune system and metabolic processes, which are closely associated with cell survival and growth. As a representative subset of the GO terms, the “interactive graph” view of REViGO on the network system of the apoptosis- and cell cycle-related genes proved that the importance mechanism of the apoptosis and cell cycles in its antitumor effect of PGL (Fig 3C).

### PGL Induces Cell Apoptosis

**Fig 4:**
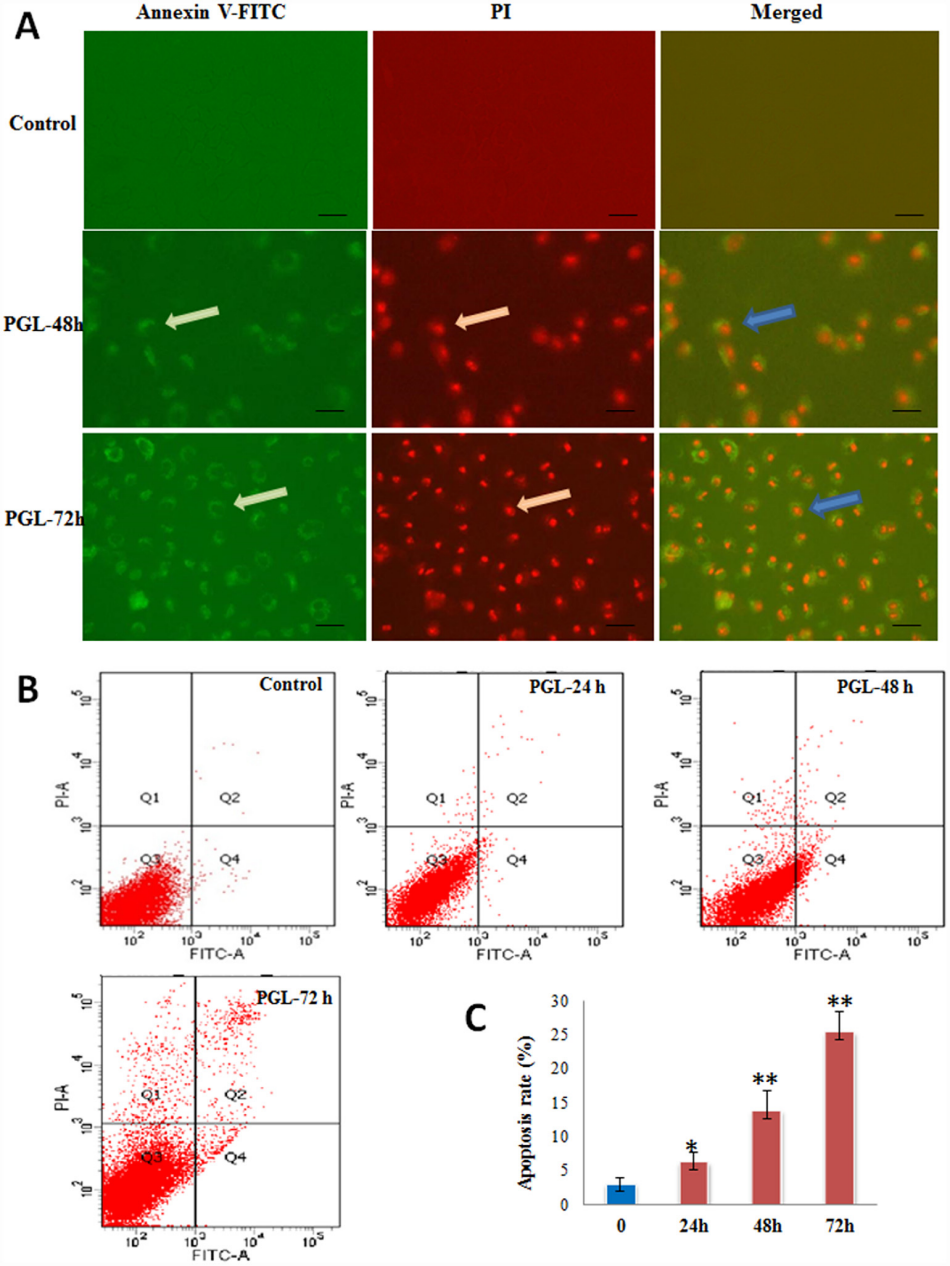
PGL induced apoptosis in A549 lung cancer cells. (A) Fluorescence-activated cell sorting analysis of Annexin V-FITC and propidium iodide (PI) for detection and quantification of PGL-induced apoptosis. Apoptosis was assessed by fluorescence microscopy after A549 cells were treated with PGL for 48 and 72 h. The photographs were taken at × 20 magnification. Scale bar 50 μm; (B, C) A549 cells were treated with PGL for 24, 48, and 72 h, then the percentage of apoptosis was determined by flow cytometry. Control means without PGL treatment. Values are expressed as the mean ± standard deviation (SD) of three independent measurements. Significant differences between the PGL-treated and control groups are indicated as * (*p* < 0.05) or ** (*p* < 0.01).

To further evaluate the involvement of apoptosis in PGL-induced lung cancer cell death, the morphological changes were examined with fluorescence microscopy. Intact cells and apoptotic cells could be distinguished by simultaneously staining with Annexin V-FITC and PI. As observed by fluorescence microscopy, the morphology of PGL-treated A549 cells was different from that of the control cells. The nucleus and cell membranes of treated cells were stained red and green, which indicated that they were in apoptosis, whereas the control cells were not (Fig 4A). We also used flow cytometry to quantitatively analyze the apoptotic cells (Fig 4B). The apoptotic rates were 5.12%, 13.74%, and 25.32% after treatment with PGL for 24, 48, and 72 h, in comparison with the rate of 3.01% for the control group (Fig 4C).

### PGL Leads to Arrest of Cell-Cycle Transition in the G1 Phase

**Fig 5:**
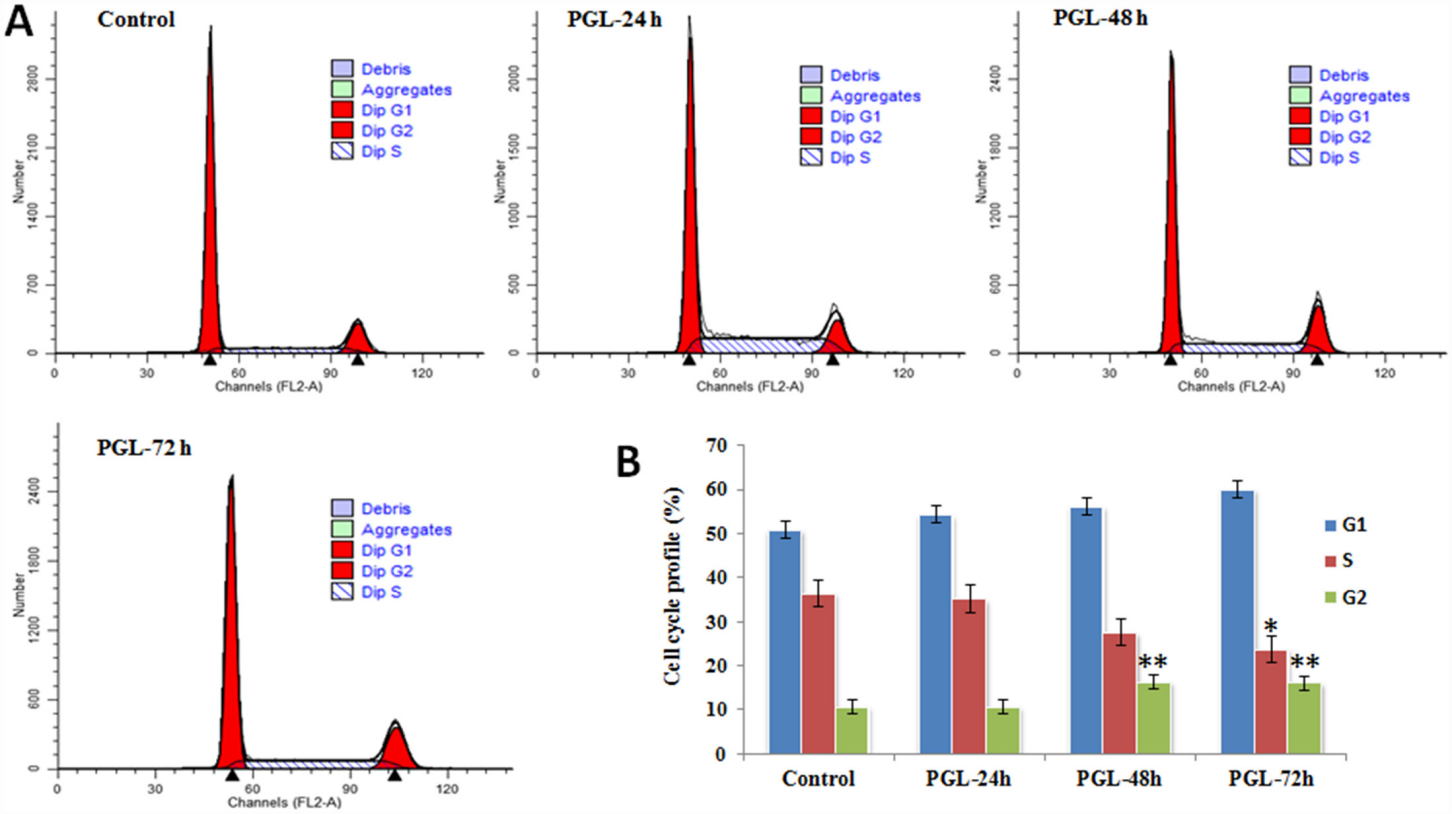
PGL changed the cell cycle distribution of A549 cells. (A) A549 cells were treated with 50 μg/mL PGL for 24, 48, and 72 h and were then harvested to determine the cell cycle distribution by flow cytometry; (B) Cell cycle profile of A549 cells after treatment with PGL for 24, 48, and 72 h. Three replicates for every time point.

To evaluate the effects of PGL on the cell cycle, A549 cells were treated with 50 μg/mL PGL for different lengths of time (24, 48, and 72 h) and flow cytometry analysis was performed on each group. PGL treatment caused arrest of the cell cycle transition in the G1 phase in a time-dependent manner (Fig 5A). Compared to the control group, the proportion of cells in the G1 phase increased significantly. The cell cycle distribution of the G1 phase with PGL (0, 24, 48, and 72 h) was 50.86%, 54.25%, 56.24%, and 60.12%, respectively. In addition, PGL-treated cells demonstrated a decrease in the population in the S phase (36.48%, 35.12%, 27.45%, and 23.79%, respectively) (Fig 5B). These results indicated that PGL could inhibit A549 cell proliferation via cell cycle arrest in the G1 phase.

### PGL Regulates the Expression of Apoptosis and Cell Cycle-Associated Genes

**Fig 6:**
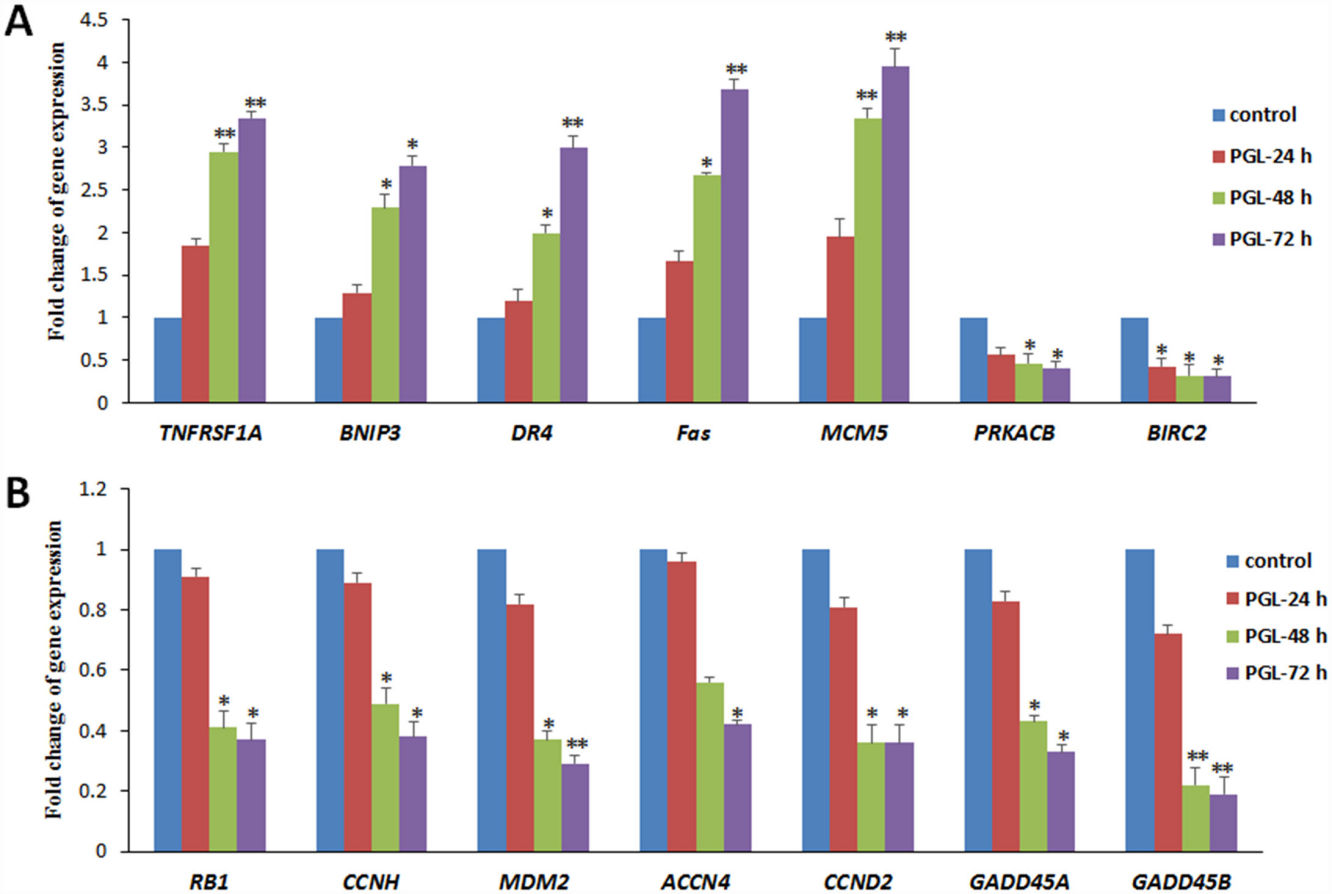
Effect of PGL on apoptosis- and cell cycle-associated mRNA levels. (A) PGL upregulated the expression of apoptosis-associated genes; (B) PGL downregulated the expression of cell cycle-associated genes. A549 cells were treated with 50 μg/mL PGL for 24, 48, and 72 h, then the mRNA levels of apoptosis- and cell cycle-related genes were examined by RT-qPCR (n = 3). Values are expressed as the mean ± SD of three independent measurements. Significant differences between the PGL-treated and control groups are indicated as * (*p* < 0.05) or ** (*p* < 0.01).

For gene sets with differential expression patterns determined by RNA-Seq analysis, we selected apoptosis- and cell cycle-related genes for further validation. We measured the mRNA levels of genes involved in apoptosis (including *TNFRSF1A*, *BNIP3*, *DR4*, *Fas*, *MCM5*, *PRKACB*, and *BIRC2*) and the cell cycle (including *RB1*, *CCNH*, *MDM2*, *ACCN4*, *CCND2*, *GADD45A*, and *GADD45B*) by RT-qPCR. We found increased mRNA levels of *TNFRSF1A*, *BNIP3*, *DR4*, *Fas*, and *MCM5*, genes involved in inducing apoptosis. In contrast, the expression levels of genes involved in inhibition of apoptosis, including *PRKACB* and *BIRC2*, were decreased (Fig 6A). Simultaneously, we observed significantly decreased mRNA levels of *RB1*, *CCNH*, *MDM2*, *ACCN4*, *CCND2*, *GADD45A*, and *GADD45B*, genes involved in cell cycle arrest (Fig 6B). We found that the expression of these genes was significantly modulated, and the expression values of these genes were consistent with RNA-Seq data (S2 Table).

## Discussion

Cancer is a major public health problem, and new drugs of all generations are necessary for its treatment. The development of resistance to chemotherapy is considered a major hindrance to the treatment of various cancers, as a notable proportion of tumors relapse and develop resistance, eventually resulting in multidrug resistance following exposure to multiple antitumor drugs with different structures and mechanisms of action [17]. Furthermore, ideal antitumor agents should act exclusively against tumor cells. However, numerous chemotherapeutic drugs presently in use exhibit significant adverse side effects (such as hair loss, diarrhea, bleeding, and immunosuppression) on the human body [18, 19]. Hence, the discovery of new natural products from plants, animals, and microorganisms that possess high efficacy against cancer cells without toxicity in normal cells is a great leap in scientific research.

There is a current tendency in the field of bioactive natural products with research applications toward a focus on marine organisms [20, 21]. More recently, compounds originating from marine algae have been found to be associated with numerous health-promoting effects, including anti-oxidative, anti-inflammatory, antiviral, antimicrobial, and antitumor effects, in particular [22, 23]. Polysaccharides of marine origin, constituting one type of these biochemical compounds, have proven to possess several important properties, such as immunomodulatory, antitumor and cancer preventive, hypoglycemic, anti-inflammatory, and antioxidant activities, making them promising bioactive products and biomaterials with a wide range of applications [4, 20]. *Gp*. *lemaneiformis* belongs to the phylum Rhodophyta, family *Gracilariaceae*, and genus *Gracilariopsis*. It possesses many bioactive functions including antitumor effects, and PGL is the key component for the antitumor activities [24]. PGL, which consists of 3,6-anhydro-L-galactose and D-galactose, is a neutral polysaccharide with a linear structure of repeated units of disaccharide agarobiose [8,23]. As a kind of natural antitumor product, polysaccharide has attracted increasing attention, and efforts in the study of polysaccharide are gradually shifting to prevention and clinical intervention in neoplastic disease [24]. Therefore, it is very important and interesting to study natural polysaccharides and their potential antitumor applications. Furthermore, the details of the antitumor mechanism remain elusive.

In the current study, we aimed to illustrate the antitumor mechanism in a genome-wide analysis of transcriptome profiles. First, we investigated the cell proliferation ability after the addition of PGL in the human gastric cancer cell line MKN45, the lung cancer cell line A549, and the cervical carcinoma cell line HeLa. The results of cell viability indicated that the antitumor effect of PGL in the A549 lung cancer cells was the most obvious by CCK-8 assay. In addition, the results of cell viability were consistent with the cell proliferation ability by trypan blue staining. On the cellular level, we found that PGL could inhibit cell proliferation ability, reduce cell viability, and alter cell morphology, and these effects were time- and concentration-dependent. Based on the anti-proliferation observation mentioned above, to understand the molecular mechanism of the PGL-induced antitumor phenotype, we first analyzed the genome-wide differentially expressed genes to identify the genes whose expression was modulated by PGL using RNA-Seq analysis. The gene expression values were calculated by Cufflinks and the distribution was also counted, and 758 differentially expressed genes were observed. To understand the biological significance of these differentially expressed genes, we annotated genes and analyzed GO using DAVID. We found that PGL affected a wide variety of biological processes, including apoptosis, the cell cycle, translation, nuclear division, and cell division. In particular, we found that PGL significantly modulated the expression of apoptosis- and cell cycle-related genes using REVIGO analysis and RT-qPCR validation.

Apoptosis in cancer cells has been shown to be the most common antitumor mechanism induced by many cancer therapies [25]. We observed increased mRNA levels of *TNFRSF1A*, which is involved in inducing apoptosis, and its protein is one of the major receptors for tumor necrosis factor-alpha [26]. We found that PGL could induce apoptosis through the activation of genes involved in the death receptor-mediated apoptosis pathway (*DR4* and *Fas*) and the p53 pathway (*MDM2*, *DR4*, and *BNIP3L*). In contrast, we observed decreased expression levels of *PRKACB* and *BIRC2*, which are associated with inhibition of apoptosis [27]. PGL regulated these genes significantly and induced apoptosis, which is the major cause of its antitumor effect.

Simultaneously, most molecular events related to the cell cycle and most antitumor drugs are designed to target particular pathways [28]. In this study, transcriptome profiling and RT-qPCR validation revealed that PGL arrests the cell cycle via downregulation of the expression of *RB1*, *CCNH*, *MDM2*, *ACCN4*, *CCND2*, *GADD45A*, and *GADD45B* in lung cancer cells. As a cell cycle regulator, *RB1* controls cell cycle progression and arrests the cell cycle by repressing E2F target genes [29]. The *GADD45A* pathway partially influences S-phase cell cycle arrest in G2 cells via cyclin D1, cyclin D3, and CDK6 [30]. These results suggest that PGL represses cell cycle-related genes and also activates apoptosis-related genes.

This study provides new insights for understanding how PGL exerts its effects against lung cancer through cellular detection of antitumor activity and genome-wide differential gene expression analysis. Apoptosis as a form of highly regulated programmed cell death has become a matter of great interest in cancer therapy; it is an important phenomenon in cytotoxicity because of the high potential that it may be induced by various chemotherapeutic agents or antitumor drugs [31]. Despite the fact that PGL is a new antitumor domain, and its theory and clinical applications should be further studied, there is no doubt that PGL appears to be one of the most promising and endless sources for drug development. Thus, it is an important task to screen marine natural products which can be used alone or in combination with other chemotherapeutic drugs to enhance the therapeutic effects and reduce the side effects in cancer therapy [32]. We believe that the development and production of antitumor drugs using our abundant algal resources holds bright prospects.

## Conclusions

PGL demonstrates antitumor efficacy and might serve as a potential antitumor agent; it inhibits cell proliferation by inducing apoptosis and cell cycle arrest in a time- and concentration-dependent manner. The transcriptome analysis provides new insights for understanding how PGL exerts its effects against lung cancer. Studies on the mechanisms of antitumor activity can offer valuable insight into theoretical and technical methods, as well as guide cancer drug research and development, so as to more effectively prevent and treat cancer.

## Supporting Information

S1 Fig The polysaccharides were purified by DEAE cellulose with chloride sodium, then the main peak (P-2) was collected and marked as PGL. (TIF)

S2 Fig Schematic diagram of differentially expressed genes involved in apoptosis by KEGG. (TIF)

S3 Fig Schematic diagram of differentially expressed genes involved in the cell cycle by KEGG. (TIF)

S4 Fig DNA replication pathway of RNA-Seq in PGL-treated group compared to control. (TIF)

S1 Table Distribution of gene expression value. (XLSX)

S2 Table Up- and downregulated genes by PGL in A549 cells. (XLSX)

S3 Table GO analysis of upregulated genes by PGL in A549 cells. (XLSX)

S4 Table GO analysis of downregulated genes by PGL in A549 cells. (XLSX)

S5 Table Primer sequences for RT-qPCR. (XLSX)
