# Supplementary figures and images for: Transcriptome Profiling Reveals the Antitumor Mechanism of Polysaccharide from Marine Algae Gracilariopsis lemaneiformis
Source: PLoS One. 2016 Jun 29;11(6):e0158279. doi: 10.1371/journal.pone.0158279 (PMC4927116; doi:10.1371/journal.pone.0158279)

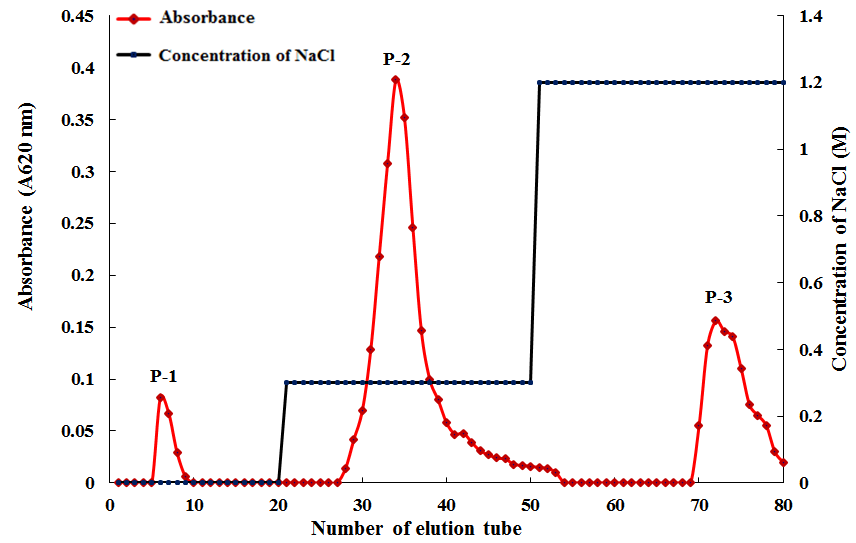

Supplement: S1 Fig — (TIF) [file pone.0158279.s001.tif]

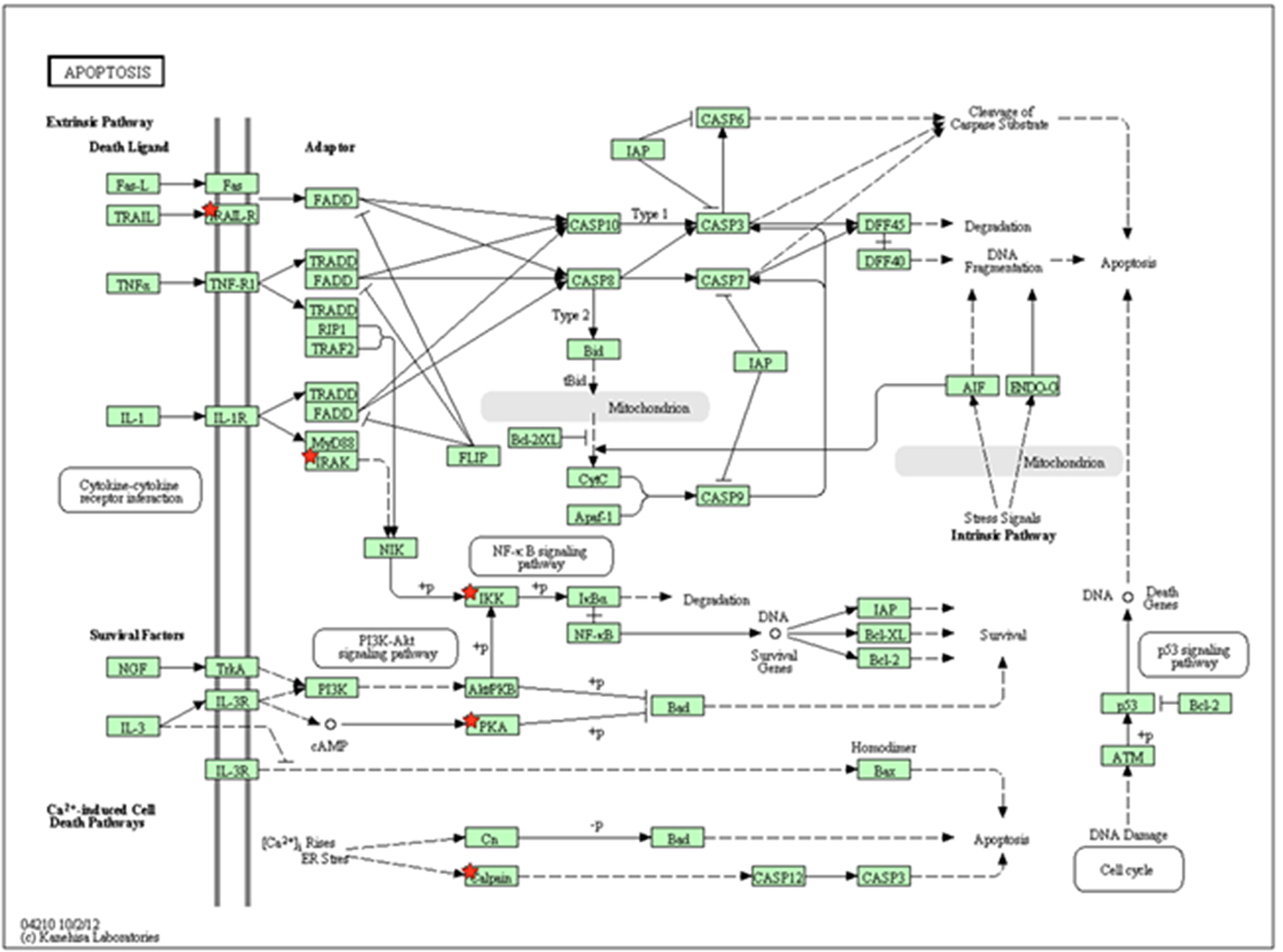

Supplement: S2 Fig — (TIF) [file pone.0158279.s002.tif]

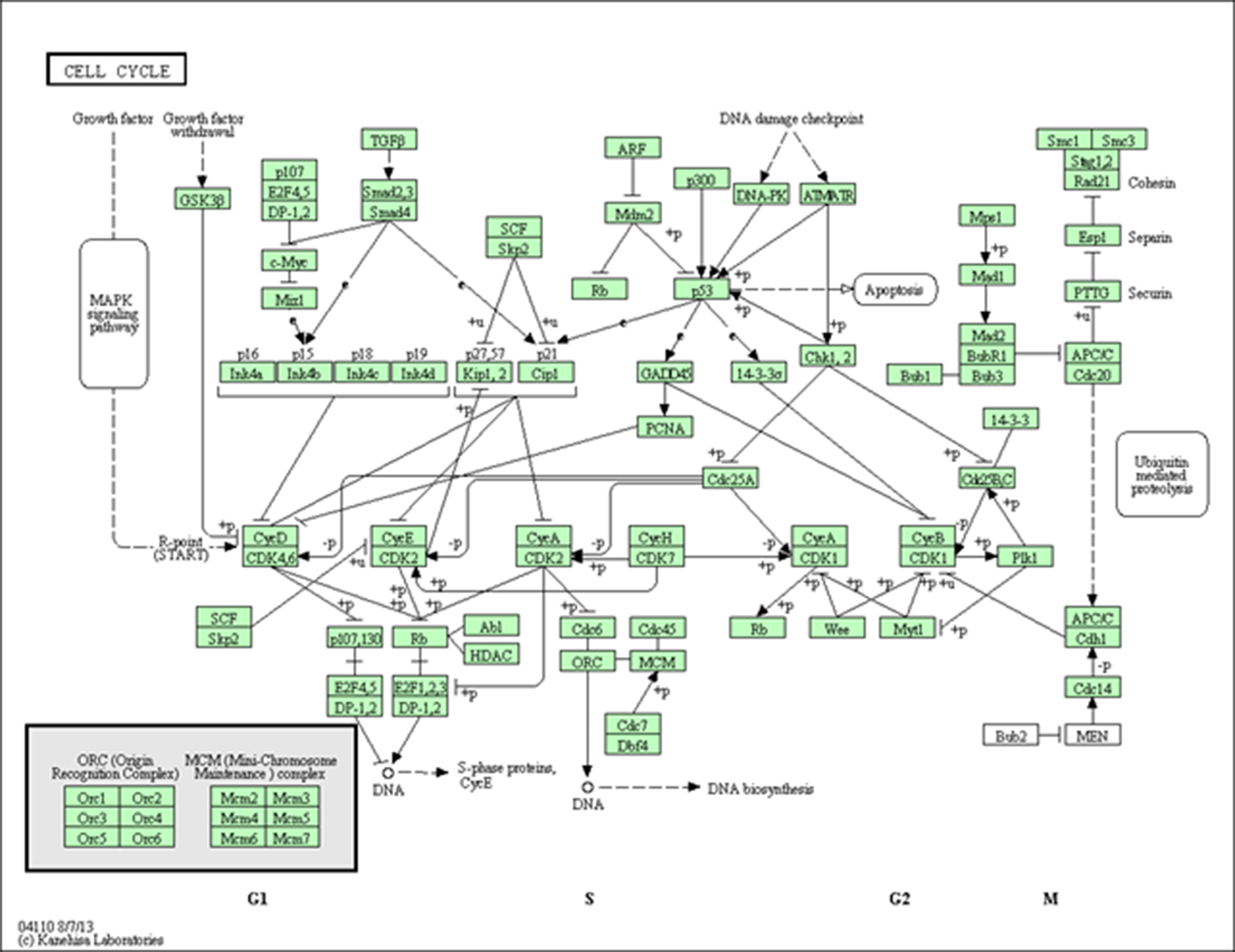

Supplement: S3 Fig — (TIF) [file pone.0158279.s003.tif]

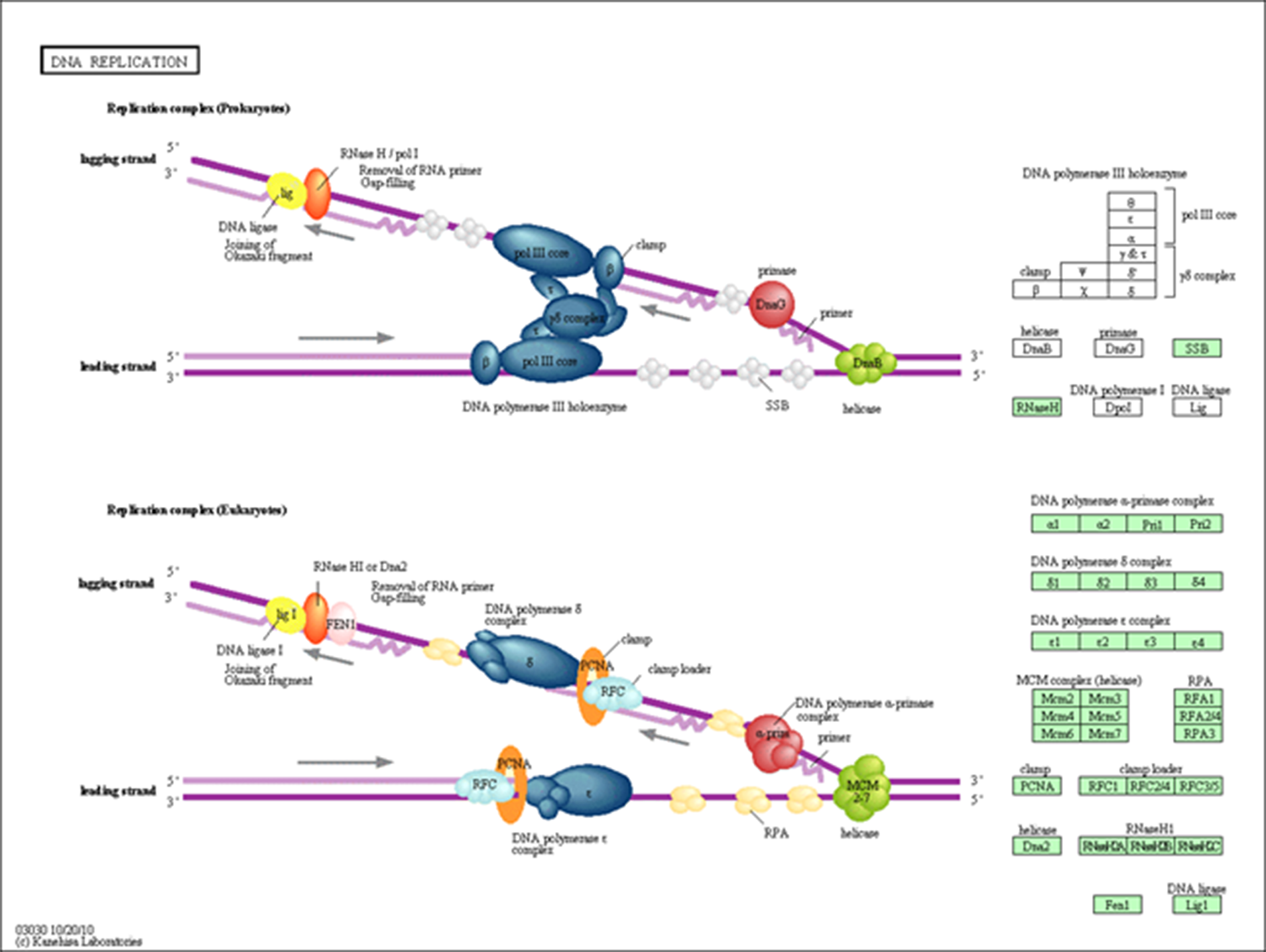

Supplement: S4 Fig — (TIF) [file pone.0158279.s004.tif]
